# Caspase 3 exhibits a yeast metacaspase proteostasis function that protects mitochondria from toxic TDP43 aggregates

**DOI:** 10.15698/mic2023.08.801

**Published:** 2023-07-10

**Authors:** Steve Brunette, Anupam Sharma, Ryan Bell, Lawrence Puente, Lynn A Megeney

**Affiliations:** 1 Regenerative Medicine Program, Sprott Centre for Stem Cell Research, Ottawa Hospital Research Institute, The Ottawa Hospital, Ottawa, ON K1H 8L6, Canada.; 2 Department of Cellular and Molecular Medicine, University of Ottawa, Ottawa, ON, Canada.; 3 Department of Medicine, University of Ottawa, Ottawa, ON, Canada.

**Keywords:** caspase, metacaspase, yeast, TDP43, proteostasis, protein aggregation

## Abstract

Caspase 3 activation is a hallmark of cell death and there is a strong correlation between elevated protease activity and evolving pathology in neurodegenerative disease, such as amyotrophic lateral sclerosis (ALS). At the cellular level, ALS is characterized by protein aggregates and inclusions, comprising the RNA binding protein TDP-43, which are hypothesized to trigger pathogenic activation of caspase 3. However, a growing body of evidence indicates this protease is essential for ensuring cell viability during growth, differentiation and adaptation to stress. Here, we explored whether caspase 3 acts to disperse toxic protein aggregates, a proteostasis activity first ascribed to the distantly related yeast metacaspase ScMCA1. We demonstrate that human caspase 3 can functionally substitute for the ScMCA1 and limit protein aggregation in yeast, including TDP-43 inclusions. Proteomic analysis revealed that disrupting caspase 3 in the same yeast substitution model resulted in detrimental TDP-43/mitochondrial protein associations. Similarly, suppression of caspase 3, in either murine or human skeletal muscle cells, led to accumulation of TDP-43 aggregates and impaired mitochondrial function. These results suggest that caspase 3 is not inherently pathogenic, but may act as a compensatory proteostasis factor, to limit TDP-43 protein inclusions and protect organelle function in aggregation related degenerative disease.

## INTRODUCTION

Pathogenic mechanisms in neurodegenerative disease have been strongly associated with disruptions in proteostasis control. One of the cardinal features of this proteostasis failure is the accumulation of toxic protein aggregates, where proteins or protein fragments accumulate in cytosolic space, overwhelming normal cell physiology and precipitating cell death [1, 2]. TAR DNA-binding protein 43 (TDP-43), an RNA binding factor, forms pathologic aggregates in all genetic and sporadic forms of amyotrophic lateral sclerosis, and is a consistent pathologic feature in frontotemporal dementia (FTD) and progressive muscle atrophy [3–5]. TDP-43 aggregation has been suggested to disrupt a plethora of cell functions, from sequestration of vital mRNAs and ribosomal machinery, to stalling proteosome activity [6–9]. Despite the variety of cell targets attributed to TDP-43, induction of apoptosis is the common end point that follows TDP-43 aggregation [10–12].

Apoptosis or caspase mediated cell death is a well-studied phenomenon, where external ligand-based signals or internal mitochondrial derived signals engage sequential caspase proteolytic cascades (extrinsic vs intrinsic mediated cell death, respectively). These signal pathways converge and terminate with the activation of effector caspases, such as caspase 3 and 7, which then target large numbers of vital protein substrates, leading to compromised cell integrity and eventual cell death [13, 14]. The intersection between protein aggregation and apoptotic signaling has led to a focus on reducing caspase activity as a tractable means to mitigate pathogenesis and cell death in TDP-43 induced proteinopathies and in aggregation induced neuropathy more broadly [15–17].

Targeting effector caspase activity as a therapeutic intervention for aggregate induced cell pathology is predicated on the concept that caspase function during neurodegeneration is exclusively destructive. However, caspase signaling has been shown to be a broadly conserved inductive cue for cell differentiation [18–21], with more recent observations suggesting that these proteases may also manage cellular adaptation to stress [22–24]. These conserved nonapoptotic activities raise an interesting supposition, that caspase activation coincident to a proteostasis challenge may be a proteolytic compensatory response, to limit or degrade aggregated proteins. The corollary to this hypothesis is that effector caspase activation transits to a true apoptotic state only when hyperactivated by unrestrained protein aggregation.

General proteostasis functions have not been described for effector caspases, yet such a biologic role may be consistent with the variety of physiologic activities attributed to these enzymes. Of note, related metacaspase proteases in yeast have been shown to be essential for limiting protein aggregation in response to stress [25, 26]. While effector caspase and metacaspase proteases are characterized by unique biochemistry, both protein types reside within the larger C14 protease clade, and share similar caspase fold structure and functional attributes [27–30]. Here, we tested whether the effector caspase, caspase 3 retained a conserved aggregate control function. We observed that human caspase 3 could functionally substitute for the single yeast metacaspase and maintain proteostasis function when challenged by overexpression of aggregating human TDP43 protein. Repression of caspase 3 activity in the heterologous yeast system resulted in a significant increase in TDP43 associations with mitochondrial proteins. This elevated TDP-43 mitochondrial protein association was also evident in murine and human skeletal muscle cells following caspase 3 inhibition and resulted in a collapse in mitochondrial function. Together, these results suggest that caspase 3 exerts a beneficial proteostasis activity, by limiting co-aggregation of toxic TDP43 with essential mitochondrial proteins.

## RESULTS

### Caspase 3 can functionally substitute for yeast ScMCA1 to maintain proteostasis

**Figure 1:**
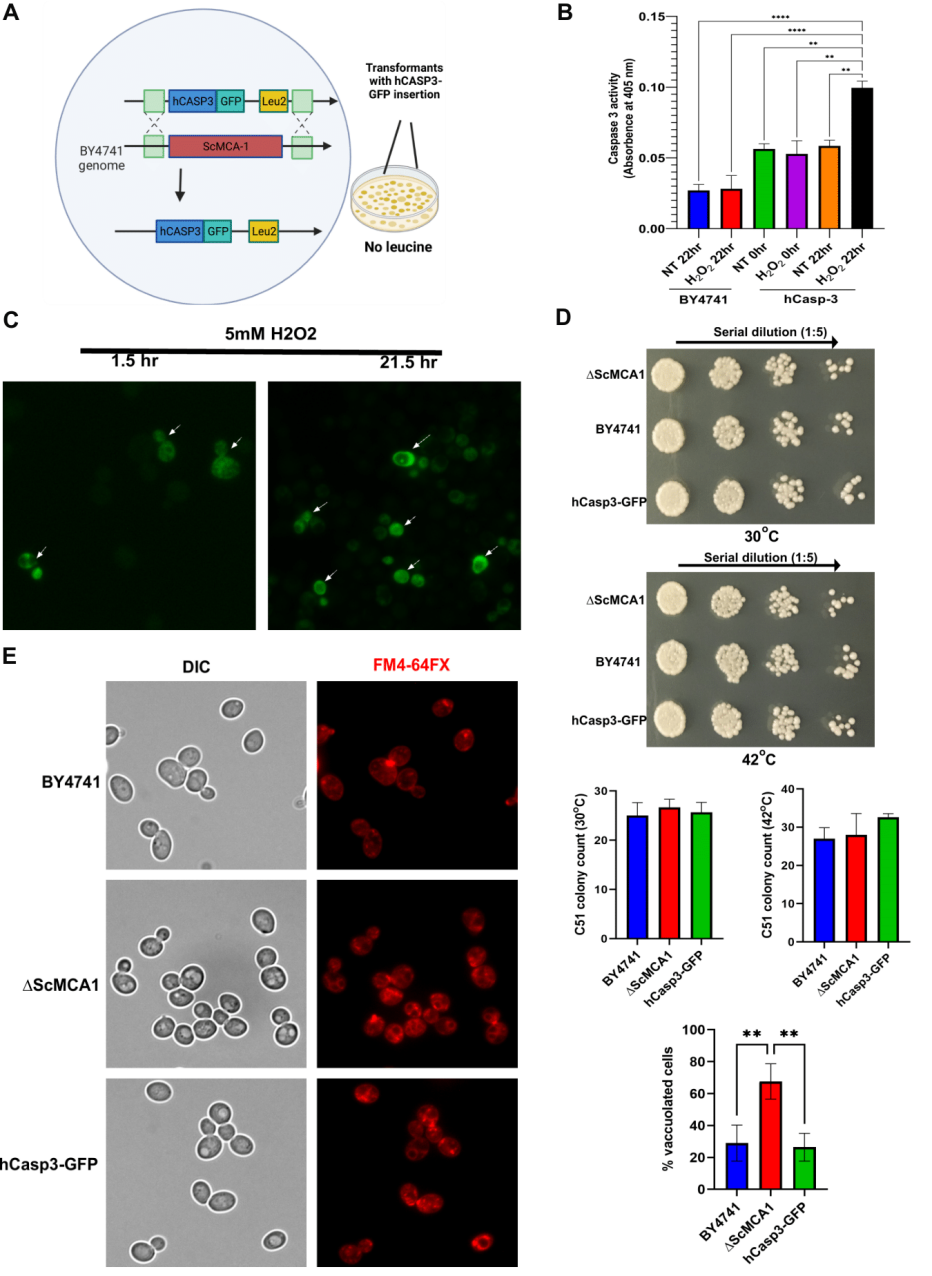
FIGURE 1: Human caspase 3 compensates for ScMCA1 activity in yeast. **(A)** Schematic representation of yeast strain with hCasp3-GFP expression in the endogenous ScMCA1 gene locus. **(B)** Colorimetry assay was performed to detect Caspase 3 activity in BY4741 and hCasp3-GFP expressing strain subjected to H_2_O_2_ induced stress. Values are mean ± SEM of three independent experiments with p-value < 0.05. **(C)** Fluorescence imaging showing hCasp3-GFP expression in yeast strain subjected to 5mM hydrogen peroxide for 1.5hr and 21.5hr. **(D)** Spot assay was performed to assess viability of three strains BY4741, Δ*ScMCA1* and hCasp3-GFP subjected to heat stress (30°C & 42°C). The arrow above the panel indicates a 5-fold serial dilution of plated cells starting from left to right (1:1, 1:5, 1:25, 1:125). The colonies were counted across the three yeast strains as indicated in the graph. The data represents three independent experiments and are shown as mean ± SEM with p-value < 0.05. **(E)** FM4-64FX staining (red) of vacuoles was performed in BY4741, Δ*ScMCA1* and hCasp3-GFP yeast strain. The red fluorescence was detected using microscopy and the images were acquired using 63X objective under oil immersion. The graph represents vacuole count across three yeast strains performed in triplicates and represented as mean ± SEM with p-value < 0.05.

Caspase and metacaspase proteases are characterized by unique biochemistry, yet both protein families share a range of biologic roles [29, 30]. The functional overlap between each protease family suggests that conserved caspase biology may be revealed using metacaspase specific organisms, where competing caspase function is absent and metacaspase activity is curtailed. To examine whether caspase 3 displayed a proteostasis function similar to the yeast metacaspase ScMCA1, we first expressed human caspase 3 in the endogenous ScMCA1 loci with a GFP tag, hCASP3-GFP (**Figure 1A**). hCASP-3-GFP yeast expressed robust levels of human caspase 3 protein, under basal and stressed conditions (Supplemental Figure S1). Utilizing this antibody (and others, data not shown) we could not identify actively processed or cleaved forms of caspase 3 (although other methods indicated that hCASP-3-GFP was subject to standard enzymatic activation, see below). Next, we measured caspase 3 enzymatic activity directly in the yeast model system. Interestingly, the hCasp-3 strains display significant elevation in caspase 3 activity compared to the parent BY strain, which is then dramatically increased in extended stress conditions such as 22 hours of H_2_O_2_ exposure (**Figure 1B**).” The observed distribution of hCASP-3-GFP in yeast cells was similar to what has been reported for ScMCA1 in both ambient and stress conditions [25, 26]. For example, the hCasp3-GFP yeast strain subjected to hydrogen peroxide induced stress displayed a robust increase in hCasp3-GFP expression at both 1.5hr and 21.5hr (**Figure 1C**). hCASP-3-GFP yeast were viable and had no alteration in cell survival compared to wildtype yeast cultures following exposure to heat stress at 42°C (as monitored by limiting dilution of cell number in a standard spot assay; **Figure 1D**). The capacity of hCASP-3-GFP to functionally substitute for ScMCA1 in yeast was evident in the ability of the human protease to maintain basic proteostasis function. Loss of ScMCA1, Δ*ScMCA1*, leads to a robust increase in autophagic vacuole formation, as a compensatory mechanism to cope with elevated insoluble protein aggregates [25]. However, the robust vacuole response in ScMCA1 null yeast was completely attenuated in the strain expressing the hCASP-3-GFP insertion from the ScMCA1 loci, suggesting that human caspase 3 can engage a proteostasis response in distantly related eukaryotic species (**Figure 1E**). In addition, these results also suggest that the pro-form of hCASP-3 may exert a proteostasis limiting function on yeast protein aggregation, which is similar to the secondary aggregate moderating effect of the ScMCA1 pro-domain [25].

### Caspase 3 can limit human TDP43 aggregation in yeast

The ability of caspase 3 to maintain yeast proteostasis implies that this protease may perform a similar function in metazoan/human cells. A reasonable extension of this hypothesis would then suggest the increased caspase 3 activity that typifies neurodegenerative disease, is simply a compensatory response to rid the affected cell of toxic insoluble proteins. For example, caspase cleavage of TDP43 has been linked to generation of toxic aggregating fragments [9, 15, 16, 31], although one study has suggested that this interaction may not be detrimental to normal cell function [32]. Therefore, we explored whether the yeast system would retain a caspase 3-TDP43 interaction, and whether this was proteostatic or proteotoxic. In addition, we reasoned that the yeast system may provide a tractable model to define conserved mechanisms by which TDP43 accumulation causes cell pathology, an issue that remains unresolved with a number of competing hypothesis [6–9].

**Figure 2:**
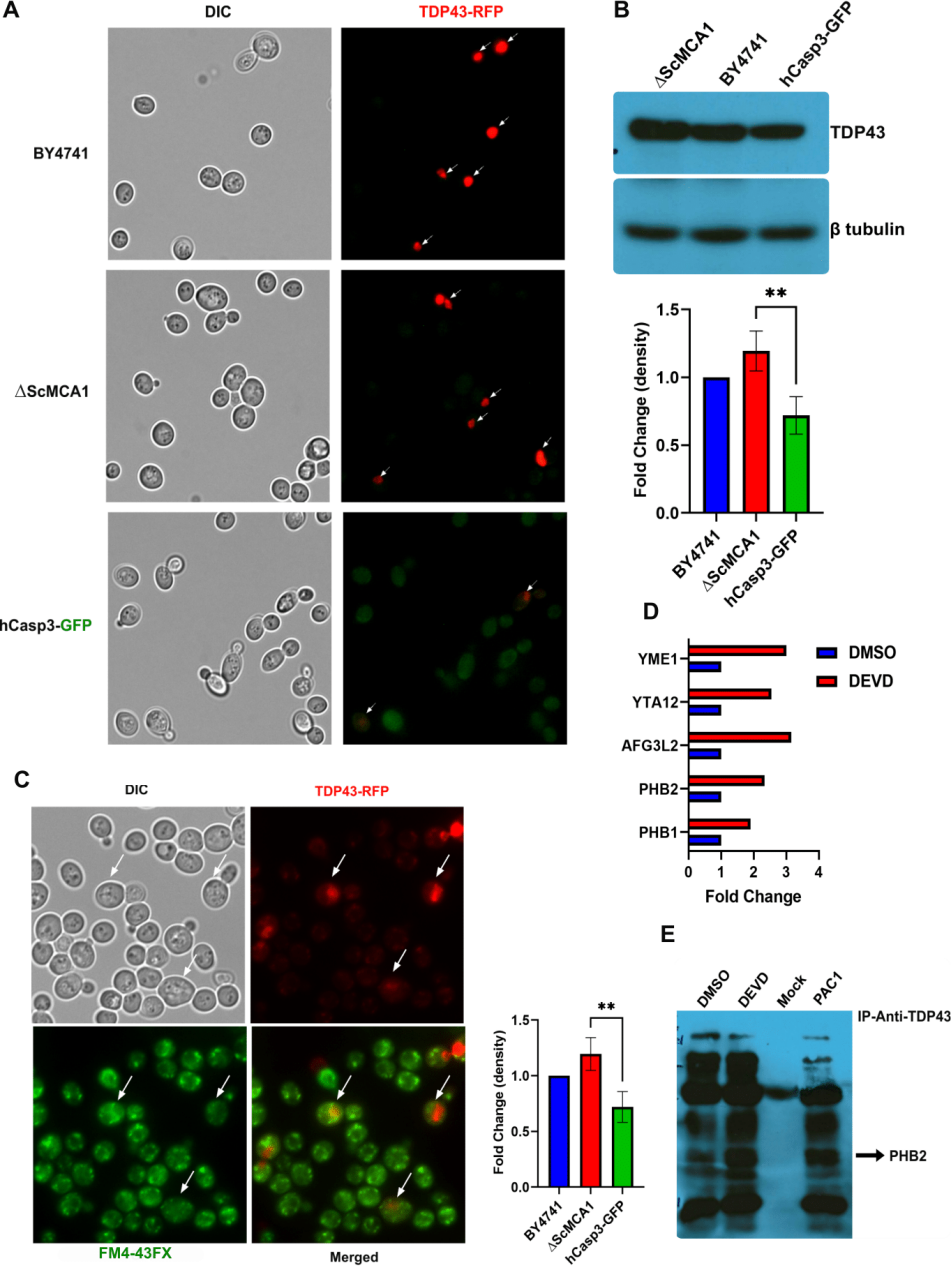
FIGURE 2: Human Caspase 3 inhibits TDP43 protein aggregation. **(A)** Representative fluorescence image showing TDP43-RFP expression (red) in three yeast strains BY4741, Δ*ScMCA1* and hCasp3-GFP. **(B)** Western blot of TDP43-RFP protein was performed in three yeast strain BY4741, Δ*ScMCA1*, and hCasp3-FP using anti-RFP antibody. The graph represents the difference in band intensities (normalized to control BY4741) across the three strains. Values are represented as mean ± SEM with p-value < 0.05. **(C)** Fluorescence image of Δ*ScMCA1* strain showing TDP-43-RFP (red) staining and vacuole stained with FM4-43FX (green). The graph shows the difference in percentage of vacuoles (≥3 vacuoles per cell) across the three yeast strains. Values are represented as mean ± SEM with p-value < 0.05. **(D)** Mass Spectrometry (MS/MS) analysis of proteins immuno-precipitated with TDP43 using anti-RFP magnetic beads in hCasp3-GFP yeast strain with and without z-DEVD-FMK treatment. The graph represents proteins with high fold interaction (normalized to DMSO control) of TDP-43 post-z-DEVD-FMK treatment. **(E)** Representative co-immunoprecipitation blot of PHB2 with TDP43 using anti-TDP43 antibody in hCasp3-GFP yeast strain treated with DMSO, z-DEVD-FMK, and AC-1 performed in triplicates.

Human TDP43 (hTDP43-RFP) was expressed from a Gal-sensitive promoter and monitored in the wildtype yeast BY4741, the Δ*ScMCA1* and the hCASP-3-GFP strains. Interestingly, the elevated baseline caspase 3 activity in the hCasp-3 strain is further augmented by the expression of the Gal-induced expression of TDP43, suggesting that caspase 3 is also activated in response to other proteostasis challenges, such as the expression of a self-aggregating protein (Supplemental Figure S2A). hTDP43-RFP was expressed throughout the cytoplasm in the BY4741 strain, forming discrete perinuclear puncta within the cell (**Figure 2A**). While hTDP43-RFP expression was similarly elevated and dispersed in the cytoplasm of the Δ*ScMCA1* strain, the hCASP-3-GFP displayed minimal levels of the TDP43 fusion protein, implying a possible caspase related processing event (**Figure 2A**). This pattern of expression was similar in protein content as performed by western blot analysis (**Figure 2B**). In addition, spot assays revealed that hTDP43-RFP expression in the various strains did not alter cell survival, suggesting that both ScMCA1 and hCASP3 can moderate TDP43 in yeast (Supplemental Figure S2B). However, hTDP43-RFP expression in the Δ*ScMCA1* strain was associated with an increase in vacuole formation compared to BY4741 and hCASP-3-GFP (**Figure 2C**) an increase in total insoluble protein aggregates (Supplemental Figure S2C), indicating that caspase 3 could effectively compensate for a proteostasis stress in yeast cells that developed in the absence of a functional metacaspase.

To define a mechanism by which hCASP3 alters TDP43 induced biology, we performed hTDP43 immunoprecipitation in the hCASP3 strain, with and without exposure to a known caspase 3 proteolysis inhibitor, the caspase 3/7 specific peptide z-DEVD-fmk, followed by MS/MS analysis to identify co-precipitating proteins. The rationale for this experiment was that any perturbation of caspase 3 function may reveal toxic TDP43 protein interactions and/or the preferred cellular protein targets for aggregating TDP43. To this end, we measured caspase 3 activity in the hCasp-3/TDP43 expressing strain subjected to the z-DEVD-FMK inhibitor and a small molecule specific activator of caspase 3, PAC1 (**P**rocaspase **A**ctivating **C**ompound 1). PAC1 is a procaspase 3 specific zinc chelating compound, which relieves zinc mediated repression of the pro-form of the enzyme. Once activated by PAC1, derepressed procaspase 3 can sequentially autoactivate pools of procaspase 3 molecules, characterized by active caspase 3 dimer formation. As such, titration of PAC1 concentration can be used to drastically elevate caspase 3 activity, and usher in fulminant apoptosis in cancer cells (100μM). Alternatively, PAC1 use at lower concentrations (∼25μM) can lead to lower levels of caspase activation which engage nondeath outcomes, such as muscle progenitor cell differentiation or cardiomyocyte hypertrophy [33–36]. Here, we noted that PAC1 treatment resulted in significantly elevated caspase 3 activity, while z-DEVD-FMK treatment led to a moderate 20% decrease in activity that did not achieve statistical significance (Supplemental Figure S2D).

Although z-DEVD-FMK treatment was unable to achieve a global reduction in yeast expressed caspase 3 activity, we reasoned that the inhibitor may be used to disrupt a specific subcellular fraction of the active protease, such as the caspase 3 pool that may target TDP43 in the yeast model. To this end we treated Gal induced caspase 3/TDP43 expressing cells with or without z-DEVD-FMK treatment and then conducted TDP43 immunoprecipitations coupled to LC-MS to identify a pool of TDP43 interacting proteins that were sensitive to caspase 3 expression and/or activity. While DMSO and z-DEVD-FMK conditions identified many overlapping protein interactions, 27 proteins were specifically upregulated in the z-DEVD-FMK condition, while 9 proteins were downregulated (as shown in volcano plot, Supplemental Figure S2E). Interestingly, 11 of the 27 upregulated protein were mitochondrial proteins, some of which include AFG3 (AFG3L2 in humans), YTA12 (SPG7 in humans), YME1 (I-AAA protease in humans), PHB1 (prohibitin 1 in humans) and PHB2 (prohibitin 2 in humans) (**Figure 2D**; Supplemental Figure S2F; **Table 1**). Of note, alterations of the human homologues for each of these 5 proteins have been shown to contribute to varied forms of neurodegeneration [37–40]. PHB2 is particularly relevant, as reductions in prohibitin expression have been linked to neurodegenerative conditions, such as Tau related aggregation/pathology [41]. Indeed, IP/western blotting confirmed an elevated interaction between TDP43 and PHB2 when hCASP3 was inhibited in yeast with z-DEVD-fmk treatment (**Figure 2E**).

**Table 1.** Mitochondrial proteins upregulated in DEVD treated group compared to DMSO control.

	**Proteins**	**Fold Change**	**p-value**
1	Mitochondrial External NADH-ubiquinone oxidoreductase **NDE1**	5.6	0.0001
2	Putative cysteine synthase **YGR012W**	5.3	0.0005
3	Mitochondrial Ketol-acid reductoisomerase **ILV5**	4.0	0.0001
4	Dihydrolipoyllysine-residue acetyltransferase component of pyruvate dehydrogenase complex, **LAT1**	2.6	0.0001
5	Mitochondrial respiratory chain complexes assembly protein **AFG3**	2.4	0.0001
6	Mitochondrial inner membrane i-AAA protease supercomplex **YMEI**	2.1	0.0096
7	Mitochondrial respiratory chain complexes assembly protein **YTA12**	1.8	0.0001
8	Prohibitin 2 **PHB2**	1.8	0.0001
9	Ubiquinol-Cytochrome C reductase complex **COR1**	1.7	0.0002
10	Mitochondrial ATP synthase subunit beta **ATP2**	1.4	0.0001
11	Prohibitin 1 **PHB1**	1.2	0.0022

### Caspase 3 activity protects mitochondrial function in skeletal muscle myocytes by limiting TDP43 aggregation

**Figure 3:**
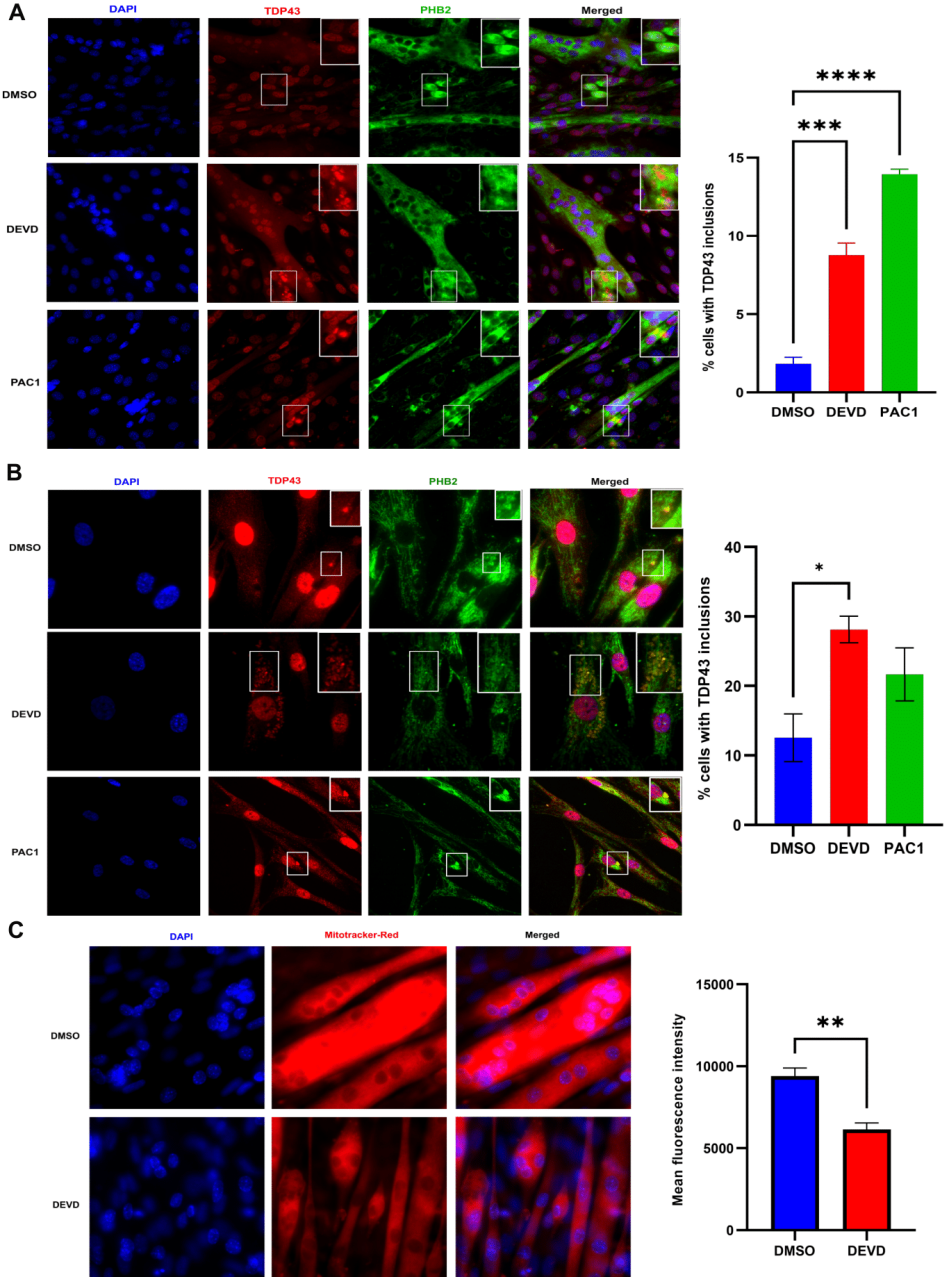
FIGURE 3: Caspase 3 inhibits TDP43 protein aggregation in skeletal muscle cells. **(A)** Representative immunofluorescence image of C2C12 cells treated with DMSO, z-DEVD-FMK and PAC1 showing protein PHB2 (green) and TDP43 (red) and their colocalization (yellow). The graph represents the percentage of TDP43 inclusions in C2C12 cells treated with DMSO, z-DEVD-FMK, and PAC1. The experiments were performed in triplicates and the values are represented as mean ± SEM with p-value < 0.05. **(B)** Representative immunofluorescence image of human primary myoblast treated with DMSO and PAC1 showing protein PHB2 (green) and TDP43 (red) and their colocalization (yellow). The graph represents the percentage of TDP43 inclusions in n primary myoblast cells treated with DMSO, z-DEVD-FMK, and PAC1. The experiments were performed in triplicates and the values are represented as mean ± SEM with p-value < 0.05 **(C)** Representative fluorescence image of C2C12 cells treated with DMSO and z-DEVD-FMK stained with MitoTracker Red CMXRos for assessment of mitochondrial function. The graph represents difference in mean fluorescence intensity of MitoTracker Red CMXRos (red) in C2C12 cells treated with DMSO and z-DEVD-FMK. The experiments were performed in triplicate with values represented as mean ± SEM with p-value < 0.05.

**Figure 4:**
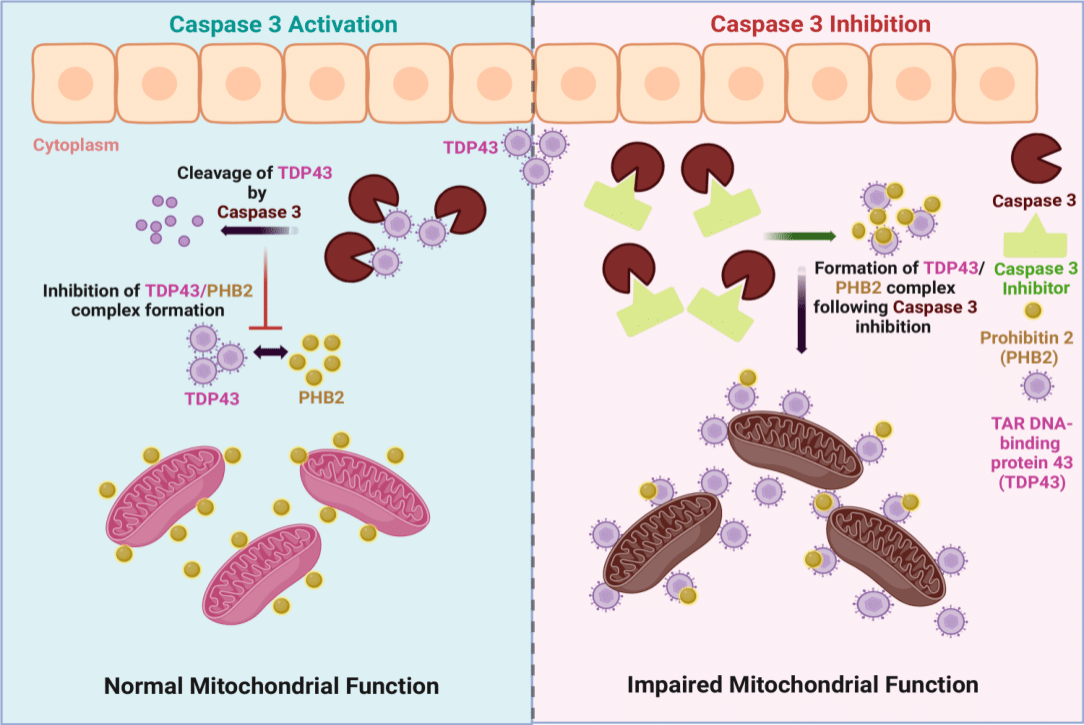
FIGURE 4: Model depicting the role of caspase 3 in proteostasis and mitochondrial function. In normal physiological state, moderate activation of caspase 3 leads to the disaggregation of TDP43 protein inhibiting its association with PHB2 leading to normal mitochondrial function. On the contrary, caspase 3 inhibition will facilitate the association of TDP43 with PHB2 which will lead to impaired mitochondrial function and can act as a driver for pathologies such as ALS.

Next, we examined whether a similar caspase 3/TDP43 relationship existed in a disease relevant cell type. Here, we used skeletal muscle cells as a tractable model, as low levels of transient TDP43 aggregation are beneficial, acting as a repository for relevant mRNA molecules that guide cell differentiation [42]. In contrast, persistent or elevated levels of aggregated TDP43 are noted in skeletal muscle fibers of ALS patients and contribute to evolving pathology of muscle tissue in animal models of ALS [43–45]. We validated caspase 3 activity in differentiated C2C12 muscle cells subjected to DMSO (control), z-DEVD-FMK and PAC1 treatment for 3hr, 6hr and 12hr following differentiation, and as anticipated, z-DEVD-FMK treatment led to significant decrease in activity at all timepoints examined. However, whereas PAC1 treatment led to a rapid and robust increase in caspase 3 activity, continued exposure to PAC1 led to a significant decline in protease activity (Supplemental Figure S3). We have noted similar responses to PAC1 treatment in fully differentiated cell types such as cardiomyocytes, where caspase 3 dependent gene expression is rapidly and robustly engaged by PAC1, followed by a sequential decrease in the same caspase 3 dependent gene expression and caspase 3 activity during continued PAC1 exposure [36]. Fully differentiated murine skeletal muscle cells that were subject to caspase 3 inhibition (z-DEVD-fmk) displayed elevated levels of TDP43 aggregates that co-localized with PHB2 (prohibitin 2) (**Figure 3A**). Similar observations were noted in fully differentiated human skeletal muscle cells, where basal caspase 3 activity was required to prevent TDP43 aggregation and colocalization with PHB2, whereas excess caspase 3 activity resulted in an exaggerated aggregation phenotype (**Figure 3B**). This suggests that caspase 3 activity must be carefully titrated, as early excessive caspase 3 activity/proteolysis itself may act as a TDP43 aggregate seeding event or to accelerate TDP43 aggregate deposition. Alternatively, PAC1 may induce the loss of an accessible pool of active caspase 3, which allows the aggregate seeding event to initiate. Irrespective of the precise mechanism by which PAC1 inspires cell pathology, early excessive caspase activation is detrimental. Mitochondrial activity was also disrupted in differentiated murine muscle cells, where inhibition (z-DEVD-fmk) of caspase 3 activity led to significant declines in mitochondrial mass and function (**Figure 3C**). Indeed, careful examination of the microscopy imaging revealed that the increased TDP43/PHB2 physical association following caspase 3 inhibition was cytoplasmic, suggesting that essential mitochondrial proteins were being sequestered in an off-target domain (**Figures 3A, 4**).

## DISCUSSION

Collectively, our observations demonstrate that moderate caspase 3 activity is required to limit the formation of toxic TDP43 aggregates (**Figure 4**). These data extend the understanding of the nonapoptotic function of effector caspases, as prospective regulatory factors in proteostasis, a role that is shared with the yeast ScMCA1 metacaspase. Caspase 3 and ScMCA1 are divergent proteins within the larger clade of caspase-like proteases, with unique biochemical characteristics. Nevertheless, caspase 3 and ScMCA1 also retain structural similarity in the caspase fold domain, suggesting that these proteases may share particular cell functions (proteostasis management) and/or substrate preferences. How caspase 3 is activated in this context remains unknown. Clearly, caspase 3 can be activated in the yeast system, yet these eukaryotic cells do not retain the sequential metazoan apoptotic signaling cascades that engage effector caspase activity. One probable mechanism of activation for caspase 3 in the yeast system may occur through proximity induced autocatalysis. This is a well described mechanism for the induction of initiator caspase activation (caspase 8, 9 and 10), where scaffolding of these enzymes through assemble of multiprotein complexes leads to elevated local concentration, allowing for dimerization and autocatalysis [13]. Effector caspase activation is considered to be the target of these scaffolding protease complexes, yet lipid raft dimerization of caspase 3 appears sufficient to induce protease activation independent of classic initiator caspase interactions [46]. In addition, procaspase 3 itself retains an autoinhibitory peptide in the loop region between the large and small subunits, which when altered has been shown to provide for a caspase 3 activation signal independent of full catalytic processing [47]. Alternatively, there is the example of PAC1 itself, as described above, PAC1 chelates inhibitory zinc ions from procaspase 3, which itself leads to activation of the pro form of the protease. This activation may then transition to a full autoactivation and cleavage processing of caspase 3, depending on the time and concentration of PAC1 exposure [33, 34]. Whether there is an operative form of biologic chelation in yeast that leads to elevated activity of human pro-caspase 3 will require further investigation. Finally, it is germane to note that our observations do not exclude a role for the pro-form of caspase 3 in the management of protein aggregation. Indeed pro and active forms of ScMCA-1 retain the capacity to manage protein aggregation, although the active form is more robust in this regard [25], suggesting that a similar division of labour may exist for caspase 3.

There has been considerable debate as to the cellular target(s) of TDP43 aggregation, yet our observations suggest that TDP43 inspired pathology originates (in part) from TDP43 driven cytoplasmic sequestration of critical mitochondrial proteins. Indeed, our observations are consistent with a prior study demonstrating a toxic association between human TDP43 and yeast mitochondria [10]. In addition, it is probable, that TDP43 may engage other pathogenic protein interactions that can be mitigated by caspase 3 activity. For example, a recent report demonstrated that TDP43 aggregates impair neuromuscular junction formation by binding to and inhibiting the translation of transcripts for nuclear encoded mitochondrial proteins [48]. Whether caspase 3 targeting of TDP43 is active in this scenario is unknown, yet a caspase 3 directed limitation of TDP43 to effect synapse maturation would be consistent with prior reports that describe an absolute requirement for this caspase protease in dendritic and axonal growth during neuronal differentiation [49–51].

Finally, it is interesting to consider how pervasive the proteostatic function is for caspase 3. Does this protease intersect with other regulatory pathways to moderate global protein turnover? Of note, the drosophila effector caspase Dcp-1, has been shown to traffic to mitochondria membranes and enhance autophagic flux through targeted suppression of SesB, an adenine nucleotide translocase that suppresses lysosomal mediated removal of cellular proteins [22]. While this is not a direct example of aggregation dissolution, this is an example of an effector caspase exerting direct control over a proteostasis related function. Secondly, it is of interest to determine whether caspase 3 beneficially targets other aggregation prone substrates to maintain cell fitness. A wide variety of proteinopathies and neurodegenerative disease are characterized by insoluble protein aggregates of varying composition, with concurrent activation of caspase 3. Our observations suggest that coincident activation of caspase 3 may not be strictly pathogenic and may simply reflect engagement of a proteostasis regulatory mechanism to disperse toxic aggregates in general. If this latter mechanism is operative, then the suggested targeting of this protease in ALS or other related neurodegenerative conditions should be approached with caution, as loss of caspase 3 activity may inadvertently lead to aggregate accumulation, exacerbating pathology rather than resolving disease progression.

## MATERIALS AND METHODS

### Yeast strains

All *Saccharomyces cerevisiae* strains used in this study were derived from the wildtype haploid BY4741 (MATa his3Δ1 leu2Δ0 met15Δ0 ura3Δ0) strain as previously described [25, 52]. The ScMCA1 strain was obtained from Open Biosystems. To generate the human procaspase 3 GFP fusion gene, an eGFP tag was amplified from ScMCA1-GFP gDNA [25] and inserted in frame as a C terminal fusion with h-procaspase 3 (plasmid pLM109), then inserted into the endogenous ScMCA1 gene locus with Leu2 as a prototrophic marker, as described previously [25]. The resulting yeast strain was YLM5000. The Gal1 promoter was amplified from BY4741 gDNA and inserted into pRP1186 (gift from Roy Parker, University of Colorado, AZ) resulting in plasmid pLM101. Human TDP43 was then amplified from HEK-293 cDNA lacking a stop codon and inserted into vector pLM101. Plasmid pLM103 (hTDP43) was then used to form combinatorial yeast expression strains on the following backgrounds YLM4006 (ScMCA1), YLM4004 (BY4741), and YLM5010 (YLM5000, h-procaspase 3). Genomic and plasmid integrants were constructed using standard LiAc transformation methods [53].

### Yeast media and growth conditions

For all experiments, yeast cultures were grown in normal YP media with glucose (2%) overnight at 30°C, before dilution to OD_660_=0.2, unless otherwise indicated. Cultures were then maintained for 3 hours incubation to ensure logarithmic growth, with the following experimental specific conditions: For immunoprecipitation MS/MS experiments, media was switched to normal YP with Galactose (2%) containing either DMSO (2μL/ml) or z-DEVD-FMK (20μM, 1009-20c, Biovision) and cell pellets were collected 5.5 hours post induction; for western blotting of procaspase 3, half of each culture was treated with hydrogen peroxide (9.8mM) for 1 hour before cell pellets were collected; for microscopy of procaspase 3-GFP, cultures were treated with hydrogen peroxide (5mM) and imaged on the microscope after 1.5 hours and 22 hours; for western blotting of TDP43, the media was switched to normal YP with Galactose (2%) and cell pellets were collected following 5.5 hours; for yeast TDP43 microscopy, the media was switched to normal YP with Galactose (2%). After 22 hours of induction the cells were fixed with 4% PFA (in PBS) for 15min at RT followed by microscopic imaging; for PHB2 co-immunoprecipitation experiments the media was switched to normal YP with Galactose (2%) for 5.5 hours containing either DMSO (2μL/ml), z-DEVD-FMK (20μM), or PAC1 (25μM, S2738, Selleck Chemicals LLC); for yeast Caspase 3 activity assay involving hydrogen peroxide treatment, half of the cultures were treated with hydrogen peroxide (5mM) and cell pellets of all cultures were collected 22 hours later; for yeast Caspase 3 activity assay with TDP43 expressed, half of the culture had media switched to normal YP with Galactose (2%) for 6 hours containing either DMSO (2μL/ml) or PAC1 (25μM) before cell pellets were collected. The corresponding YP with glucose (2%) culture was treated with either DMSO (2uL/ml) or PAC1 (25μM) and cell pellets were collected 6 hours later.

### Yeast spot assays

Yeast cultures were grown as above. For strains without TDP43 expression the culture concentrations were determined by spectrophotometer and then diluted to an OD_660_=0.1. Then 200μL was added to 800μL sterile ddH2O for C1 (1280 cells/5μL). 3 more serial dilutions of 1:5 were performed (C2=256 cells, C3=51 cells, C4=10 cells). 5μL of each dilution were then spotted onto a YPD agar plate. Once spots were dried one plate was placed in the humidified 30°C incubator. For HS conditions one plate was placed into the humidified 42°C incubator for 2 hours and then placed in 30°C incubator. All plates were incubated for 48 hours then photographed. For strains containing the TDP43 expression plasmid, the YPD agar plates were substituted with YPGR agar plates. Photos were quantified using Image J.

### Yeast vacuole stains

Cultures were grown in acidic YP (pH3.5) with glucose (2%) overnight at 30°C, before dilution to OD660=0.2. After 3 hours of incubation to attain logarithmic growth phase, the cultures were pelleted and resuspended in 2ml's of acidic YPD containing FM4-64FX (17μM, F34653, Invitrogen). After 40min of staining the cells were fixed with 4% PFA (in PBS) for 15min at RT followed by microscopic imaging (25,28). Fluorescence was detected by microscopic observation using a Zeiss Observer Z1, with a Plan Apochromat 63X objective under oil immersion with a 1.0X camera adapter. Red fluorescence (emission, 599nm) images were used to score the percentage of cells per field displaying vacuoles (one or more).

Cultures were grown in YP with glucose (2%) overnight at 30°C, before dilution to OD_660_=0.2. After 3 hours of incubation to attain logarithmic growth phase, the media was switched to normal YP with Galactose (2%) for 22 hours. The cultures were then pelleted and resuspended in 2ml's of YP with Galactose (2%) containing FM1-43FX (18μM, F35355, Invitrogen). After 40min of staining the cells were fixed with 4% PFA (in PBS) for 15min at RT followed by microscopic imaging [25, 28]. Fluorescence was detected by microscopic observation using a Zeiss Observer Z1, with a Plan Apochromat 63X objective under oil immersion with a 1.0X camera adapter. Green fluorescence (emission, 488nm) images were used to score the percentage of cells per field displaying vacuoles (three or more).

### Yeast western blots and co-immunoprecipitations

Protein was extracted in modified RIPA buffer (50mM Tris-HCl, 1% Nonidet P-40 substitute, 150mM NaCl, 1mM EDTA, and 1% glycerol supplemented with 20μg/mL each aprotinin, pepstatin, leupeptin, and PMSF) through glass-bead disruption, separated by PAGE, and blotted onto a PVDF membrane. Immunodetection was performed using anti-Procaspase 3 1:1000 (ab32499, Abcam), for TDP43-RFP, anti-RFP 1:2000 (M155-3; MBL Medical Biological Laboratories Co.), and anti–β-tubulin 1:25 (E7, Developmental Hybridoma Ban, Iowa State University) primary antibodies. Secondary antibodies were as follows, goat anti-Rb-HRP 1:10,000 (170-6515, BioRad) to detect Pro-caspase 3, and goat anti-Ms-HRP 1:10,000 (170-6516, Bio-Rad) to detect TDP43-RFP or tubulin were applied.

For PHB2 co-immunoprecipitation (co-IP), proteins were extracted in IP buffer (20mM HEPES pH7.4, 2mM MgCl2, 300mM NaCl, 0.1% Tween-20 supplemented with 20μg/mL each aprotinin, pepstatin, leupeptin, and PMSF) through glass-bead disruption. Co-IP was accomplished using 500μg of total protein extract (volumes normalized) with 5μL anti-TDP43 (ab190963, Abcam) and 50μL anti-Rb Dynabeads (11203D, Invitrogen). Following separation by PAGE, and blotting onto a PVDF membrane, immunodetection of PHB2 was performed using anti-REA 1:1000 (ab181838, Abcam) primary antibody and secondary antibody goat anti-Rb-HRP 1:10,000 (170-6515, Bio-Rad).

### Mass spectrometry analysis of yeast TDP43 co-immunoprecipitation

Protein was extracted in IP buffer as indicated above. Co-IP was accomplished using 875μg of total protein extract (volumes normalized) with 50μL anti-RFP magnetic beads, directed against TDP43-RFP (M165-11, MBL Medical Biological Laboratories Co.). Following separation by PAGE and silver staining, individual lanes were divided into 5 equal fragments and digested in-gel using trypsin (Promega) according to the method of Shevchenko [54]. The resulting peptide extracts were concentrated by vacufuge (Eppendorf) then dissolved in 1% formic acid (Fisher).

Peptides were analyzed by LC-MS/MS (liquid chromatography tandem mass spectrometry) using an UltiMate 3000 RSLC nano HPLC, an LTQ Orbitrap XL hybrid mass spectrometer, and a nanospray ionization source and Xcalibur software version 2.0.7 (Thermo Scientific). Samples were loaded by autosampler onto a C18 trap column (Thermo) in 3% acetonitrile, 0.1% formic acid and washed at a flow rate of 15 microlitres per minute for 5 minutes. Peptides were eluted over a 60-minute gradient of 3% - 45% acetonitrile at a flow rate of 300 nanolitres per minute to a 10-cm long column with integrated emitter tip (Picofrit PF360-75-15-N-5 from New Objective packed with Zorbax SB-C18, 5 micron from Agilent), and nanosprayed into the mass spectrometer. MS scans were acquired in FTMS mode at a resolution setting of 60,000. MS^2^ scans were acquired in ion trap CID mode using data-dependent acquisition of the top 5 ions from each MS scan.

MASCOT software version 2.5 (Matrix Science, UK) was used to infer peptide and protein identities from the mass spectra. The observed MS/MS spectra were matched against human and yeast sequences from SwissProt (version 2014-08) and also against a database of common contaminants. Mass tolerance parameters were MS ±10 ppm and MS/MS ±0.6 Da. Enzyme specificity was set to ‘Trypsin' with <=2 miscuts. Oxidation of methionine, protein N-terminal acetylation, pyrocarbamidomethlyation of N-terminal cysteine, and conversion of glutamine to pyroglutamate were allowed as variable modifications. Carbamidomethylation of cysteine was set as a fixed modification.

### Muscle cell culture

Cells of the immortalized murine skeletal muscle cell line, C2C12, were grown on non-collagen coated cell culture plates in Dulbecco's Modified Eagle's medium (DMEM) with 10% fetal bovine serum (FBS) and 1% penicillin/streptomycin (growth medium). Once confluent, the cells were differentiated in DMEM supplemented with 2% horse serum and 1% penicillin/streptomycin (differentiation medium). Human primary myoblasts were isolated, as described [55]. Following isolation, myoblasts were cultured in 1:1 (v/v) of Ham's F10:DMEM, supplemented with 20% FBS, 1% penicillin/streptomycin, and 10 ng/mL basic fibroblast growth factor on collagen coated coverslips (see below). Myoblasts were differentiated in DMEM supplemented with 5% horse serum and 1% penicillin/streptomycin. For immunofluorescence experiments, muscle cells were grown on coverslips with or without collagen as indicated (Fisherbrand glass coverslips 1.0 thickness, 25mm circles). For assessing the caspase activity in C2C12s, the cells were differentiated for 48 hours prior to treatment with z-DEVD-FMK and PAC1. The cells were treated with either DMSO (2μL/ml) as a negative control, z-DEVD-FMK (20μM), and PAC1 (25μM). Differentiated samples were collected before treatment and then 3, 6, and 12 hours later.

### Muscle cell immunofluorescence

Following low serum induction of differentiation (post 48 hours), C2C12 cells and human primary myoblasts were treated with either DMSO (2μL/ml) as a negative control, z-DEVD-FMK (20μM), or a 3-hour pulse of PAC1 (25μM). Treatments were refreshed at four days post low serum induction of differentiation. After six days of differentiation conditions, cells were fixed with 4% PFA (in PBS) for 10min at RT, then treated with 0.3% Triton X100 (in PBS) for 10 min at RT. TDP43 was detected using anti-TDP43 1:500 (ab104223, Abcam) and PHB2 using anti-REA 1:250 (ab181838, Abcam). Secondary antibodies used were goat anti-mouse Alexa 568 1:2000 (A11031, Invitrogen) to detect TDP43, and goat anti-rabbit Alexa 488 1:2000 (A11008, Invitrogen) to detect PHB2. Cells were also co-stained with DAPI (20ng/ml in PBS) for 10min at RT. Coverslips were prepped for microscopy using DAKO mounting medium. The resulting fluorescence was detected by microscopy using a Zeiss Observer Z1, with a Pan Apochromat objective 20X, or 63X under oil immersion with a 0.63X camera adapter. TDP43 inclusions were counted visually.

### Mitochondrial membrane potential

Mitochondrial membrane potential was evaluated in live myotubes by use of MitoTracker Red CMXRos (M7512, Invitrogen). Following low serum induction of differentiation (post 48 hours), C2C12 cells were treated with either DMSO (2μL/ml) as a negative control, or z-DEVD-FMK (20μM). Treatments were refreshed at four days post low serum induction of differentiation. At six days cells were treated with MitoTracker (200nM) for 30min at 37°C. Cells were then fixed with 4% PFA (in PBS) for 10 min at RT. They were then treated with 0.3% Triton X100 (in PBS) for 10min at RT and then stained with DAPI (10ng/ml in PBS) for 10min at RT and mounted on a slide using DAKO mounting medium. The resulting fluorescence was detected by microscopic observation using a Zeiss Observer Z1, with a 40X Pan Apochromat objective under oil immersion with a 0.63X camera adapter. Red fluorescence (emission, 599nm) was quantified by use of ImageJ software.

### Caspase 3 activity assay

Caspase 3 activity was determined using the colorimetry based Caspase-3 Assay Kit (ab39401) according to the manufacturer”s instruction. Briefly, protein was isolated from yeast and C2C12 cells and the concentration was determined using the Bradford assay. 100μg of protein lysate was incubated with a specific substrate, Ac-DEVD-pNA at 37°C for 4 hours, and caspase activity was measured spectrophotometrically at 405nm.

### Filter trap assay for insoluble protein quantification

Filter trap assay was conducted using the same conditions and insoluble protein concentrations as previously described [25]. Quantification was performed using Image J.

### Differential protein expression analysis

The proteomic mass spectra data obtained from MS/MS analysis were visualised using Scaffold (version Scaffold_4.8.4, Proteome Software Inc., Portland, OR). For protein identification to be considered valid, it needed to meet the following criteria: a protein threshold 99%, a peptide threshold of 95% and at least one identified peptide. Fischer's exact test with multiple testing by Benjamini-Hochberg method with an adjusted p-value of 0.05 was used to extract significantly enriched proteins.

### Statistical Analysis

The statistical analysis was performed using GraphPad Prism version 9.4.1. The statistical significance between the means of the three yeast strains was evaluated using the one-way ANOVA with Tukey's and Bonferroni's multiple comparison test. Student t-test was performed for analysis of MitoTracker Red CMXRos relative fluorescence intensity measurements. All the experiments were performed in triplicates and p-value < 0.05 was considered to indicate a statistically significant difference.

## SUPPLEMENTAL MATERIAL

Click here for supplemental data file.

All supplemental data for this article are available online at www.microbialcell.com/researcharticles/2023a-brunette-microbial-cell/.

## Abbreviations:

ALS: – amyotrophic lateral sclerosis,
TDP-43: – TAR DNA-binding protein 43,
FTD: – frontotemporal dementia,
PAC1: – Procaspase Activating Compound 1,
PHB2: – prohibitin 2.

## References

[1] Ben-Gedalya T, Moll L, Bejerano-Sagie M, Frere S, Cabral WA, Friedmann-Morvinski D, Slutsky I, Burstyn-Cohen T, Marini JC, Cohen E (2015). Alzheimer's disease-causing proline substitutions lead to presenilin 1 aggregation and malfunction.. The EMBO Journal.

[2] Klaips CL, Jayaraj GG, Hartl FU (2017). Pathways of cellular proteostasis in aging and disease.. Journal of Cell Biology.

[3] Kabashi E, Valdmanis PN, Dion P, Spiegelman D, McConkey BJ, Vande Velde C, Bouchard J-P, Lacomblez L, Pochigaeva K, Salachas F, Pradat P-F, Camu W, Meininger V, Dupre N, Rouleau GA (2008). TARDBP mutations in individuals with sporadic and familial amyotrophic lateral sclerosis.. Nat Genet.

[4] Johnson BS, Snead D, Lee JJ, McCaffery JM, Shorter J, Gitler AD (2009). TDP-43 Is Intrinsically Aggregation-prone, and Amyotrophic Lateral Sclerosis-linked Mutations Accelerate Aggregation and Increase Toxicity *.. Journal of Biological Chemistry.

[5] McAlary L, Plotkin SS, Yerbury JJ, Cashman NR (2019). Prion-Like Propagation of Protein Misfolding and Aggregation in Amyotrophic Lateral Sclerosis.. Frontiers in Molecular Neuroscience.

[6] Lagier-Tourenne C, Polymenidou M, Hutt KR, Vu AQ, Baughn M, Huelga SC, Clutario KM, Ling S-C, Liang TY, Mazur C, Wancewicz E, Kim AS, Watt A, Freier S, Hicks GG, Donohue JP, Shiue L, Bennett CF, Ravits J, Cleveland DW, Yeo GW (2012). Divergent roles of ALS-linked proteins FUS/TLS and TDP-43 intersect in processing long pre-mRNAs.. Nat Neurosci.

[7] Ling JP, Pletnikova O, Troncoso JC, Wong PC (2015). TDP-43 repression of nonconserved cryptic exons is compromised in ALS-FTD.. Science.

[8] Bjork RT, Mortimore NP, Loganathan S, Zarnescu DC (2022). Dysregulation of Translation in TDP-43 Proteinopathies: Deficits in the RNA Supply Chain and Local Protein Production.. Frontiers in Neuroscience.

[9] Riemenschneider H, Guo Q, Bader J, Frottin F, Farny D, Kleinberger G, Haass C, Mann M, Hartl FU, Baumeister W, Hipp MS, Meissner F, Fernández-Busnadiego R, Edbauer D (2022). Gel-like inclusions of C-terminal fragments of TDP-43 sequester stalled proteasomes in neurons.. EMBO reports.

[10] Braun RJ, Sommer C, Carmona-Gutierrez D, Khoury CM, Ring J, Büttner S, Madeo F (2011). Neurotoxic 43-kDa TAR DNA-binding Protein (TDP-43) Triggers Mitochondrion-dependent Programmed Cell Death in Yeast *.. Journal of Biological Chemistry.

[11] Lucini CB, Braun RJ (2021). Mitochondrion-Dependent Cell Death in TDP-43 Proteinopathies.. Biomedicines.

[12] Sreedharan J, Blair IP, Tripathi VB, Hu X, Vance C, Rogelj B, Ackerley S, Durnall JC, Williams KL, Buratti E, Baralle F, de Belleroche J, Mitchell JD, Leigh PN, Al-Chalabi A, Miller CC, Nicholson G, Shaw CE (2008). TDP-43 Mutations in Familial and Sporadic Amyotrophic Lateral Sclerosis.. Science.

[13] Fuchs Y, Steller H (2011). Programmed Cell Death in Animal Development and Disease.. Cell.

[14] Connolly PF, Jäger R, Fearnhead HO (2014). New roles for old enzymes: killer caspases as the engine of cell behavior changes.. Frontiers in Physiology.

[15] Zhang Y-J, Xu Y-F, Cook C, Gendron TF, Roettges P, Link CD, Lin W-L, Tong J, Castanedes-Casey M, Ash P, Gass J, Rangachari V, Buratti E, Baralle F, Golde TE, Dickson DW, Petrucelli L (2009). Aberrant cleavage of TDP-43 enhances aggregation and cellular toxicity.. Proc Natl Acad Sci USA.

[16] Hart MP, Gitler AD (2012). ALS-Associated Ataxin 2 PolyQ Expansions Enhance Stress-Induced Caspase 3 Activation and Increase TDP-43 Pathological Modifications.. J Neurosci.

[17] Moujalled D, Strasser A, Liddell JR (2021). Molecular mechanisms of cell death in neurological diseases.. Cell Death Differ.

[18] Fernando P, Kelly JF, Balazsi K, Slack RS, Megeney LA (2002). Caspase 3 activity is required for skeletal muscle differentiation.. Proceedings of the National Academy of Sciences.

[19] Arama E, Agapite J, Steller H (2003). Caspase Activity and a Specific Cytochrome C Are Required for Sperm Differentiation in Drosophila.. Developmental Cell.

[20] Fujita J, Crane AM, Souza MK, Dejosez M, Kyba M, Flavell RA, Thomson JA, Zwaka TP (2008). Caspase Activity Mediates the Differentiation of Embryonic Stem Cells.. Cell Stem Cell.

[21] Bell RAV, Megeney LA (2017). Evolution of caspase-mediated cell death and differentiation: twins separated at birth.. Cell Death Differ.

[22] DeVorkin L, Go NE, Hou Y-CC, Moradian A, Morin GB, Gorski SM (2014). The Drosophila effector caspase Dcp-1 regulates mitochondrial dynamics and autophagic flux via SesB.. Journal of Cell Biology.

[23] Weaver BP, Weaver YM, Omi S, Yuan W, Ewbank JJ, Han M (2020). Non-Canonical Caspase Activity Antagonizes p38 MAPK Stress-Priming Function to Support Development.. Developmental Cell.

[24] Conde-Rubio M del C, Mylonas R, Widmann C (2021). The proteolytic landscape of cells exposed to non-lethal stresses is shaped by executioner caspases.. Cell Death Discov.

[25] Lee REC, Brunette S, Puente LG, Megeney LA (2010). Metacaspase Yca1 is required for clearance of insoluble protein aggregates.. Proceedings of the National Academy of Sciences.

[26] Hill SM, Hao X, Liu B, Nyström T (2014). Life-span extension by a metacaspase in the yeast *Saccharomyces cerevisiae*.. Science.

[27] Wong AH-H, Yan C, Shi Y (2012). Crystal Structure of the Yeast Metacaspase Yca1.. Journal of Biological Chemistry.

[28] Shrestha A, Brunette S, Stanford WL, Megeney LA (2019). The metacaspase Yca1 maintains proteostasis through multiple interactions with the ubiquitin system.. Cell Discov.

[29] Minina EA (2020). Classification and Nomenclature of Metacaspases and Paracaspases: No More Confusion with Caspases.. Molecular Cell.

[30] Carmona-Gutierrez D, Fröhlich K-U, Kroemer G, Madeo F (2010). Metacaspases are caspases. Doubt no more.. Cell Death Differ.

[31] Li Q, Yokoshi M, Okada H, Kawahara Y (2015). The cleavage pattern of TDP-43 determines its rate of clearance and cytotoxicity.. Nat Commun.

[32] Suzuki H, Lee K, Matsuoka M (2011). TDP-43-induced Death Is Associated with Altered Regulation of BIM and Bcl-xL and Attenuated by Caspase-mediated TDP-43 Cleavage *.. Journal of Biological Chemistry.

[33] Putt KS, Chen GW, Pearson JM, Sandhorst JS, Hoagland MS, Kwon J-T, Hwang S-K, Jin H, Churchwell MI, Cho M-H, Doerge DR, Helferich WG, Hergenrother PJ (2006). Small-molecule activation of procaspase-3 to caspase-3 as a personalized anticancer strategy.. Nat Chem Biol.

[34] Peterson QP, Goode DR, West DC, Ramsey KN, Lee JJY, Hergenrother PJ (2009). PAC-1 Activates Procaspase-3 in Vitro through Relief of Zinc-Mediated Inhibition.. Journal of Molecular Biology.

[35] Dick SA, Chang NC, Dumont NA, Bell RAV, Putinski C, Kawabe Y, Litchfield DW, Rudnicki MA, Megeney LA (2015). Caspase 3 cleavage of Pax7 inhibits self-renewal of satellite cells.. Proceedings of the National Academy of Sciences.

[36] Putinski C, Abdul-Ghani M, Stiles R, Brunette S, Dick SA, Fernando P, Megeney LA (2013). Intrinsic-mediated caspase activation is essential for cardiomyocyte hypertrophy.. Proceedings of the National Academy of Sciences.

[37] Chiang H-L, Fuh J-L, Tsai Y-S, Soong B-W, Liao Y-C, Lee Y-C (2021). Expanding the phenotype of AFG3L2 mutations: Late–onset autosomal recessive spinocerebellar ataxia.. Journal of the Neurological Sciences.

[38] Ferreirinha F, Quattrini A, Pirozzi M, Valsecchi V, Dina G, Broccoli V, Auricchio A, Piemonte F, Tozzi G, Gaeta L, Casari G, Ballabio A, Rugarli EI (2004). Axonal degeneration in paraplegin-deficient mice is associated with abnormal mitochondria and impairment of axonal transport.. J Clin Invest.

[39] Casari G, De Fusco M, Ciarmatori S, Zeviani M, Mora M, Fernandez P, De Michele G, Filla A, Cocozza S, Marconi R, Dürr A, Fontaine B, Ballabio A (1998). Spastic Paraplegia and OXPHOS Impairment Caused by Mutations in Paraplegin, a Nuclear-Encoded Mitochondrial Metalloprotease.. Cell.

[40] Merkwirth C, Martinelli P, Korwitz A, Morbin M, Brönneke HS, Jordan SD, Rugarli EI, Langer T (2012). Loss of Prohibitin Membrane Scaffolds Impairs Mitochondrial Architecture and Leads to Tau Hyperphosphorylation and Neurodegeneration.. PLOS Genetics.

[41] Guyot A-C, Leuxe C, Disdier C, Oumata N, Costa N, Roux GL, Varela PF, Duchon A, Charbonnier JB, Herault Y, Pavoni S, Galons H, Andriambeloson E, Wagner S, Meijer L, Lund AK, Mabondzo A (2020). A Small Compound Targeting Prohibitin with Potential Interest for Cognitive Deficit Rescue in Aging mice and Tau Pathology Treatment.. Sci Rep.

[42] Vogler TO, Wheeler JR, Nguyen ED, Hughes MP, Britson KA, Lester E, Rao B, Betta ND, Whitney ON, Ewachiw TE, Gomes E, Shorter J, Lloyd TE, Eisenberg DS, Taylor JP, Johnson AM, Olwin BB, Parker R (2018). TDP-43 and RNA form amyloid-like myo-granules in regenerating muscle.. Nature.

[43] Mori F, Tada M, Kon T, Miki Y, Tanji K, Kurotaki H, Tomiyama M, Ishihara T, Onodera O, Kakita A, Wakabayashi K (2019). Phosphorylated TDP-43 aggregates in skeletal and cardiac muscle are a marker of myogenic degeneration in amyotrophic lateral sclerosis and various conditions.. Acta Neuropathologica Communications.

[44] Lynch E, Semrad T, Belsito VS, FitzGibbons C, Reilly M, Hayakawa K, Suzuki M (2019). C9ORF72-related cellular pathology in skeletal myocytes derived from ALS-patient induced pluripotent stem cells.. Disease Models & Mechanisms.

[45] Crociara P (2019). Motor neuron degeneration, severe myopathy and TDP-43 increase in a transgenic pig model of SOD1-linked familiar ALS.. Neurobiology of Disease.

[46] Secinaro MA, Fortner KA, Dienz O, Logan A, Murphy MP, Anathy V, Boyson JE, Budd RC (2018). Glycolysis promotes caspase-3 activation in lipid rafts in T cells.. Cell Death Dis.

[47] Roy S, Bayly CI, Gareau Y, Houtzager VM, Kargman S, Keen SLC, Rowland K, Seiden IM, Thornberry NA, Nicholson DW (2001). Maintenance of caspase-3 proenzyme dormancy by an intrinsic “safety catch” regulatory tripeptide.. Proceedings of the National Academy of Sciences.

[48] Altman T, Ionescu A, Ibraheem A, Priesmann D, Gradus-Pery T, Farberov L, Alexandra G, Shelestovich N, Dafinca R, Shomron N, Rage F, Talbot K, Ward ME, Dori A, Krüger M, Perlson E (2021). Axonal TDP-43 condensates drive neuromuscular junction disruption through inhibition of local synthesis of nuclear encoded mitochondrial proteins.. Nat Commun.

[49] Fernando P, Brunette S, Megeney LA (2005). Neural stem cell differentiation is dependent upon endogenous caspase 3 activity.. FASEB J.

[50] Kanuka H, Kuranaga E, Takemoto K, Hiratou T, Okano H, Miura M (2005). Drosophila caspase transduces Shaggy/GSK-3β kinase activity in neural precursor development.. The EMBO Journal.

[51] Ohsawa S, Hamada S, Kuida K, Yoshida H, Igaki T, Miura M (2010). Maturation of the olfactory sensory neurons by Apaf-1/caspase-9–mediated caspase activity.. Proceedings of the National Academy of Sciences.

[52] Lee REC, Puente LG, Kærn M, Megeney LA (2008). A Non-Death Role of the Yeast Metacaspase: Yca1p Alters Cell Cycle Dynamics.. PLOS ONE.

[53] Gietz RD, Schiestl RH (2007). High-efficiency yeast transformation using the LiAc/SS carrier DNA/PEG method.. Nat Protoc.

[54] Shevchenko A, Tomas H, Havli J, Olsen JV, Mann M (2006). In-gel digestion for mass spectrometric characterization of proteins and proteomes.. Nat Protoc.

[55] Feige P, Tsai EC, Rudnicki MA (2021). Analysis of human satellite cell dynamics on cultured adult skeletal muscle myofibers.. Skeletal Muscle.

