# Supplementary material for: Caspase 3 exhibits a yeast metacaspase proteostasis function that protects mitochondria from toxic TDP43 aggregates
Source: Microb Cell. 2023 Jul 10;10(8):157–69. doi: 10.15698/mic2023.08.801 (PMC10399456; doi:10.15698/mic2023.08.801)
Supplement: Supplementary file 1 [file mic-10-157-s01.pdf]

Supplemental Information - Caspase 3 exhibits a yeast metacaspase proteostasis function that protects mitochondria from toxic TDP43 aggregates

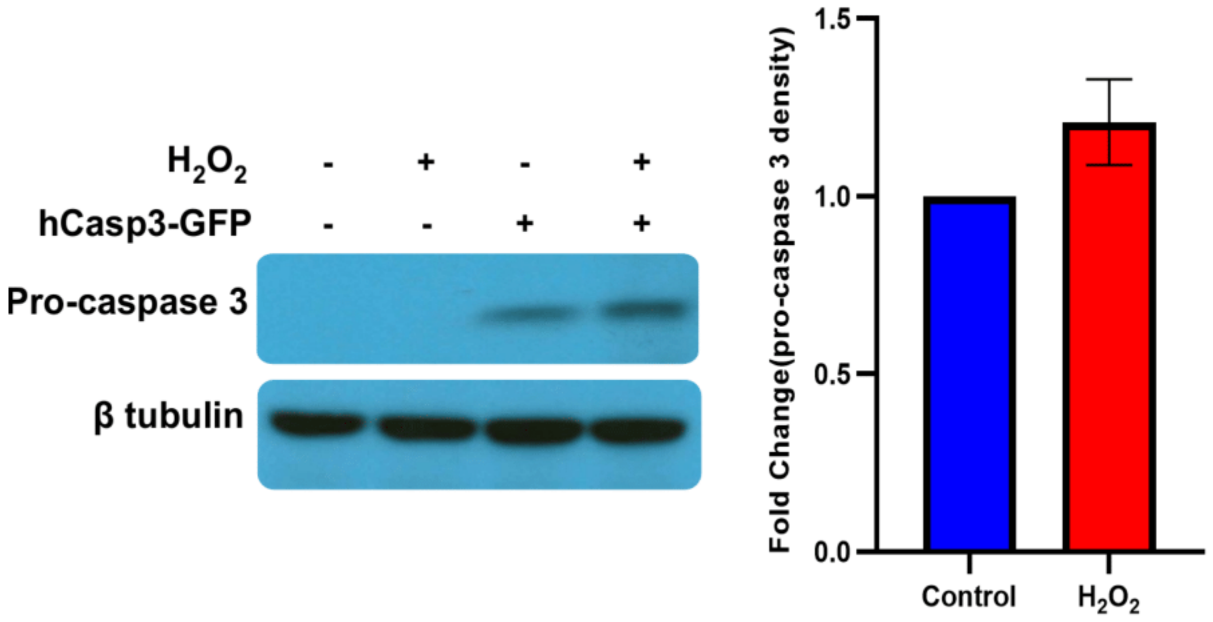

FIGURE S1: Western blot analysis of protein extract from BY4741 and hCasp3-GFP showing robust procaspase 3 protein expression following hydrogen peroxide treatment. The graph shows the quantification of the band intensity normalized to the control group. Values are mean ± SEM of three independent experiments with p-value < 0.05.

S2A

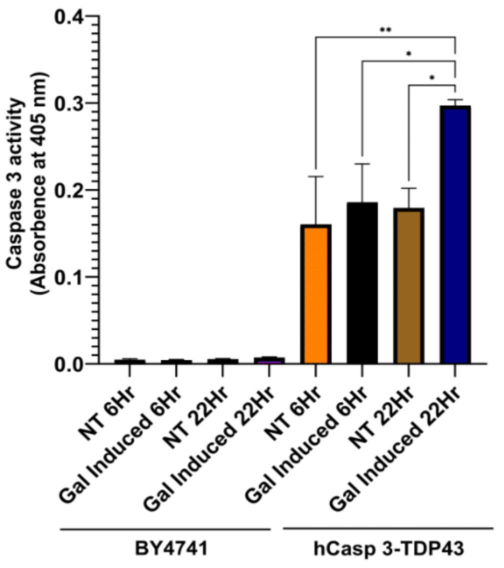

S2B

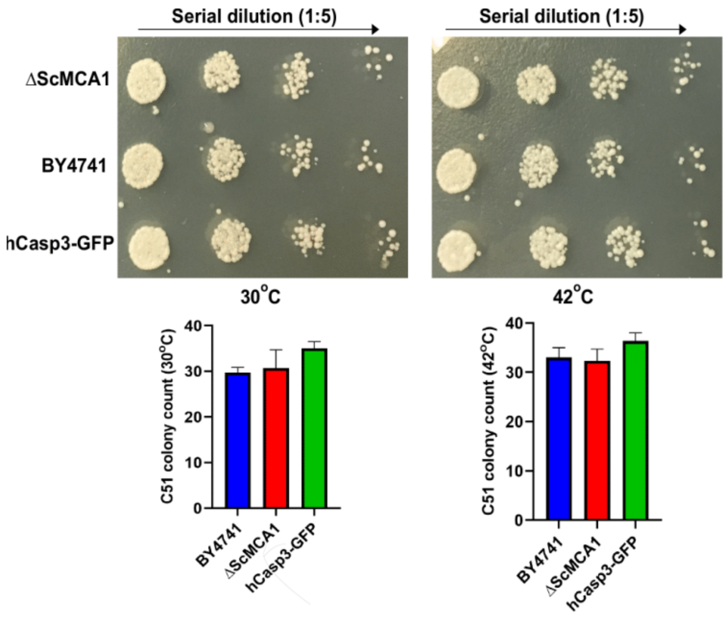

S2C

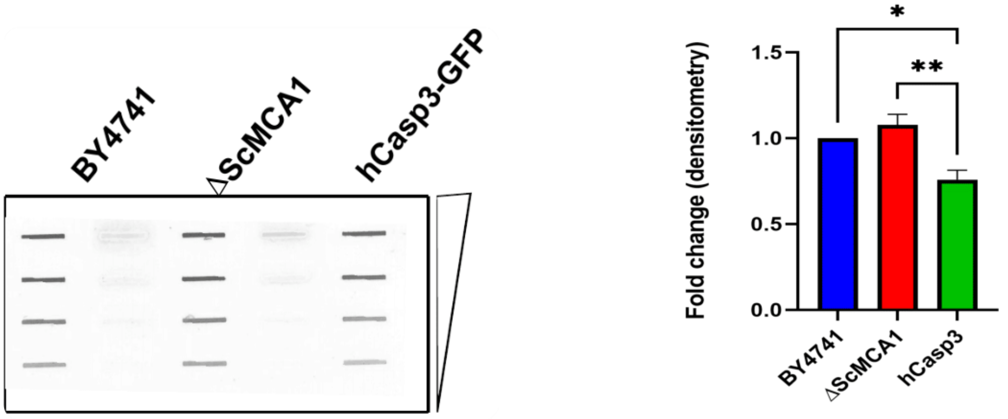

S2D

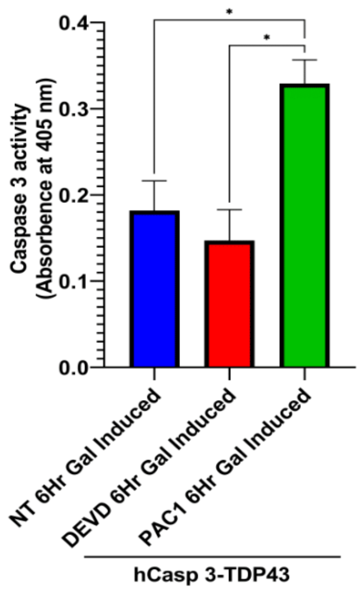

S2E

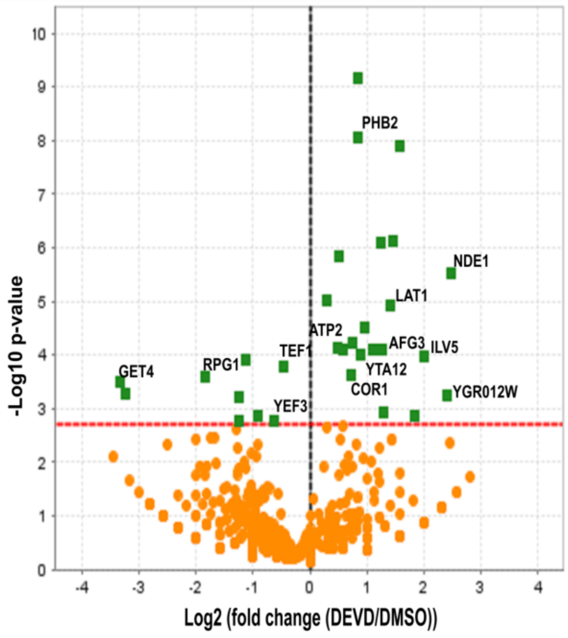

S2F

P40961 (100%), 31,427.9 Da
Prohibitin-1 OS=Saccharomyces cerevisiae (strain ATCC 204508 / S288c) GN=PHB1 PE=1 SV=2
7 exclusive unique peptides, 13 exclusive unique spectra, 14 total spectra, 120/287 amino acids (42% coverage)

```
MSNSAKLIDV ITKVALPIGI IASGIQYSMY DVKGGSRGVI FDRINGVKQQ VVGEGTHFLV
PWLQKAIIYD VRTKPKSIAT NTGTKDLQMV SLTLRVLHRP EVLQLPAIYQ NLGLDYDERV
LPSIGNEVLK SIVAQFDAAE LITQREIISQ KIRKELSTRA NEFGIKLEDV SITHMTFGPE
FTKAVEQKQI AQQDAERAKF LVEKAEQERQ ASVIRAEGEA ESAEFISKAL AKVGDGLLLI
RRLEASKDIA QTLANSSNVV YLPSQHSGGG NSESSGSPNS LLLNIGR
```

P50085 (100%), 34,407.2 Da
Prohibitin-2 OS=Saccharomyces cerevisiae (strain ATCC 204508 / S288c) GN=PHB2 PE=1 SV=2
2 exclusive unique peptides, 2 exclusive unique spectra, 2 total spectra, 57/310 amino acids (18% coverage)

```
MNRSPGEFQR YAKAFQKQLS KVQQTGGRGQ VPSPRGAFAG LGGLLLLGGG ALFINNALFN
VDGGHRAIVY SRIHGVSSRI FNEGTHFIFP WLDTPIIYDV RAKPRNVASL TGTKDLQMVN
ITCRVLSRPD VVQLPTIYRT LGQDYDERVL PSIVNEVLKA VVAQFNASQL ITQREKVSRL
IRENLVRRAS KFNILLDDVS ITYMTFSPEF TNAVEAKQIA QQDAQRAAFV VDKARQEKQG
MVVRAQGEAK SAELIGEAIK KSRDYVELKR LDTARDIAKI LASSPNRVIL DNEALLLNTV
VDARIDGRGK
```

P39925 (100%), 84,547.4 Da
Mitochondrial respiratory chain complexes assembly protein AFG3 OS=Saccharomyces cerevisiae (strain ATCC 204508 / S288c) GN=AFG3 PE=1 SV=1
1 exclusive unique peptides, 1 exclusive unique spectra, 2 total spectra, 31/761 amino acids (4% coverage)

```
MMMWQRYARG APRSLTSLSF GKASRISTVK PVLRSRMPVH QRLQTLSGLA TRNTIHRSTQ
IRSFHISWTR LNENRPNKEG EGKNNGNKDN NSNKEDGKDK RNEFGSLSEY FRSKEFANTM
FLTIGFTIIF TLLTPSSNNS GDDSNRVLTF QDFKTKYLEK GLVSKIYVVN KFLVEAELVN
TKQVVSFTIG SVDIFEEQMD QIQDLLNIPP RDRIPIKYIE RSSPFTFLFP FLPTIILLGG
LYFITRKINS SPPNANGGGG GGLGGMFNVG KSRAKLFNKE TDIKISFKNV AGCDEAKQEI
MEFVHFLKNP GKYTKLGAKI PRGAILSGPP GTGKTLLAKA TAGEANVPFL SVSGSEFVEM
FVGVGASRVR DLFTQARSMA PSIIFIDEID AIGKERGKGG ALGGANDERE ATLNQLLVEM
DGFTTSDQVV VLAGTNRPDV LDNALMRPGR FDRHIQIDSP DVNGRQQIYL VHLKRLNLDP
LLTDDMNNLS GKLATLTPGF TGADIANACN EAALIAARHN DPYITIHHFE QAIERVIAGL
EKKTRVLSKE EKRSVAYHEA GHAVCGWFLK YADPLLKVSI IPRGQGALGY AQYLPPDQYL
ISEEQFRHRM IMALGGRVSE ELHFPSVTSG AHDDFKKVTQ MANAMVTSLG MSPKIGYLSF
DQNDGNFKVN KPFSNKTART IDLEVKSIVD DAHRACTELL TKNLDKVDLV AKELLRKEAI
TREDMIRLLG PRPFKERNEA FEKYLDPKSN TEPPEAPAAT N
```

P40341 (100%), 93,280.1 Da
Mitochondrial respiratory chain complexes assembly protein YTA12 OS=Saccharomyces cerevisiae (strain ATCC 204508 / S288c) GN=YTA12 PE=1 SV=2
3 exclusive unique peptides, 3 exclusive unique spectra, 3 total spectra, 36/825 amino acids (4% coverage)

```
MLLLSWSRIA TKVVRRPVRF RSYYGLTHIK SLHTQYRLLN RLQENKSGNK NEDNNEDAKL
NKEIPTDEEV EAIRKQVEKY IEQTKNNTIP ANWKEQKRKI DESIRRLEDA VLKQESNRIQ
EERKEKEEEN GPSKAKSNRT KEQGYFEGNN SRNIPPPPPP PPPKPPLNDP SNPVSKNVNL
FQIGLTFFLL SFLLDLLNSL EEQSEITWQD FREKLLAKGY VAKLIVVNKS MVKVMLNDNG
KNQADNYGRN FYYFTIGSID SFEHKLQKAQ DELDIDKDFR IPVLYVQEGN WAKAMFQILP
TVLMIAGIIW LTRRSAQAAG GSRGGIFGLS RSKAKKFNTE TDVKIKFKDV AGCDEAKEEI
MEFVSFLKEP SRYEKMGAKI PRGAILSGPP GTGKTLLAKA TAGEAGVPFY FVSGSEFVEM
FVGVGAARVR DLFKTARENA PSIVFIDEID AIGKARQKGN FSGANDEREN TLNQMLVEMD
GFTPADHVVV LAGTNRPDIL DKALLRPGRF DRHINIDKPE LEGRKAIFAV HLHHLKLAGE
IFDLKNRLAA LTPGFSGADI ANVCNEAALI AARSDEDAVK LNHFEQAIER VIGGVERKSK
LLSPEEKKVV AYHEAGHAVC GWYLKYADPL LKVSIIPRGQ GALGYAQYLP GDIFLLTEQQ
LKDRMTMSLG GRVSEELHFP SVTSGASDDF KKVTSMATAM VTELGMSDKI GWVNYQKRDD
SDLTKPFSDE TGDIIDSEVY RIVQECHDRC TKLLKEKAED VEKIAQVLLK KEVLTREDMI
DLLGKRPFPE RNDAFDKYLN DYETEKIRKE EEKNEKRNEP KPSTN
```

P32795 (100%), 81,774.2 Da
Mitochondrial inner membrane i-AAA protease supercomplex subunit YME1 OS=Saccharomyces cerevisiae (strain ATCC 204508 / S288c) GN=YME1 PE=1 SV=1
2 exclusive unique peptides, 2 exclusive unique spectra, 2 total spectra, 32/747 amino acids (4% coverage)

```
MNVSKILVSP TVTTNVLRIF APRLPQIGAS LLVQKKWALR SKKFYRFYSE KNSGEMPPKK
EADSSGKASN KSTISSIDNS QPPPPSNTND KTKQANVAVS HAMLATREQE ANKDLTSPDA
QAAFYKLLLQ SNYPQYVVSR FETPGIASSP ECMELYMEAL QRIGRHSEAD AVRQNLLTAS
SAGAVNPSLA SSSSNQSGYH GNFPSMYSPL YGSRKEPLHV VVSESTFTVV SRWVKWLLVF
GILTYSFSEG FKYITENTTL LKSSEVADKS VDVAKTNVKF DDVCGCDEAR AELEEIVDFL
KDPTKYESLG GKLPKGVLLT GPPGTGKTLL ARATAGEAGV DFFFMSGSEF DEVYVGVGAK
RIRDLFAQAR SRAPAIIFID ELDAIGGKRN PKDQAYAKQT LNQLLVELDG FSQTSGIIII
GATNFPEALD KALTRPGRFD KVVNVDLPDV RGRADILKHH MKKITLADNV DPTIIARGTP
GLSGAELANL VNQAAVYACQ KNAVSVDMSH FEWAKDKILM GAERKTMVLT DAARKATAFH
EAGHAIMAKY TNGATPLYKA TILPRGRALG ITFQLPEMDK VDITKRECQA RLDVCMGGKI
AEELIYGKDN TTSGCGSDLQ SATGTARAMV TQYGMSDDVG PVNLSENWES WSNKIRDIAD
NEVIELLKDS EERARRLLTK KNVELHRLAQ GLIEYETLDA HEIEQVCKGE KLDKLKTSTN
TVVEGPDSDE RKDIGDDKPK IPTMLNA
```

FIGURE S2: (**A**) Colorimetry assay was performed to determine the Caspase 3 activity in BY4741 and hCasp3-TDP43 expressing strain with Gal induced expression of TDP43-RFP for 6hr and 22hr. The value represents three independent experiments and are shown as mean ± SEM with p-value < 0.05. (**B**) Caspase 3 activity assay in hCasp3-TDP43 expressing strain subjected to treatment with z-DEVD-FMK and PAC1. The values correspond to mean ± SEM with p-value < 0.05. (**C**) Spot assay to assess the viability of three yeast strains BY4741, Δ*ScMCA1* and hCasp3-GFP subjected to heat stress (30°C & 42°C). The arrow above the panel indicates a 5-fold serial dilution of plated cells starting from left to right (1:1, 1:5, 1:25, 1:125). The colonies were counted and showed no difference in viability between the three yeast strains as indicated in the graph. The data represents three independent experiments and are shown as mean ± SEM with p-value < 0.05. (**D**) Protein aggregation observed in BY4741, Δ*ScMCA1* and hCasp3-GFP strain using filter trap assay. The graph shows fold difference in protein density normalized to the control BY4741. The values are represented as mean ± SEM with p-value < 0.05. (**E**) Volcano plot showing protein change in DMSO Vs. z-DEVD-FMK treated yeast cells. Protein differentially regulated with a p-value < 0.05 are shown in green. Grey represents proteins that were not found to differ significantly between z-DEVD-FMK treatment and DMSO control. The horizontal axis indicates log 2-fold change of protein abundance while the vertical axis shows -log 10 of the p-value. (**F**) Coverage of five TDP43 interacting proteins PHB1, PHB2, AFG3, YTA12 and YME1 identified in the LC-MS/MS mass spectrometry analysis.

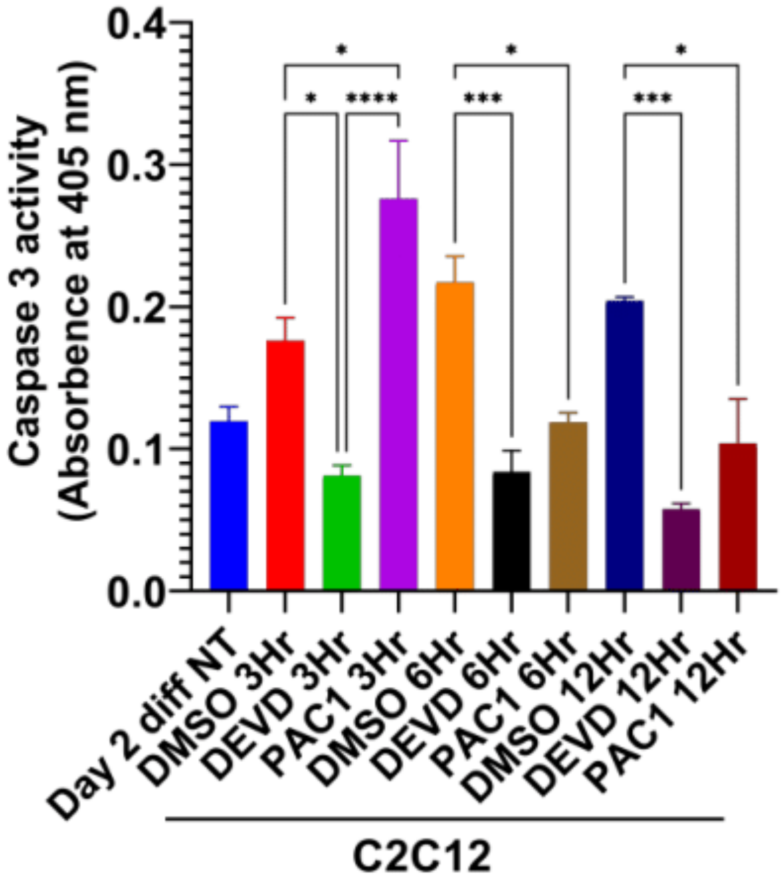

FIGURE S3: Substrate based colorimetry assay was performed to assess the Caspase 3 activity in C2C12 cells differentiated for 2 days and subjected to z-DEVD-FMK and PAC1 treatment for 3hr, 6hr & 12hr. The data is represented as mean ± SEM with p-value < 0.05.
